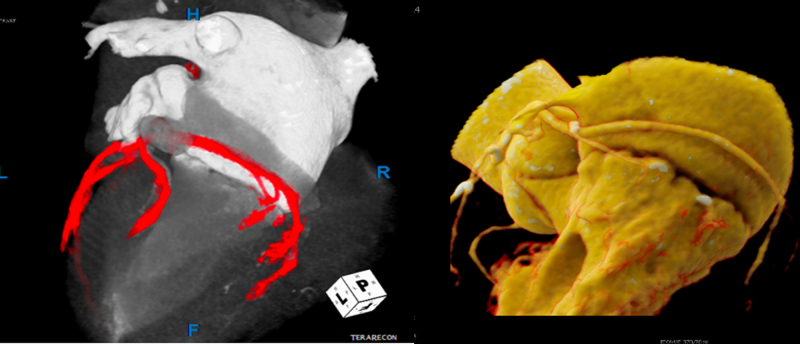# Occlusion of Abnormal Circumflex Coronary Artery During Mitral Valve Repair

**DOI:** 10.1016/j.atssr.2024.01.007

**Published:** 2024-02-08

**Authors:** Michael Dorsey, Les James, Shashwat Shrivastava, Didier Loulmet, Eugene Grossi

**Affiliations:** 1 Department of Cardiothoracic Surgery, New York University Langone Medical Center, New York, New York

## Abstract

We describe a rare but interesting complication of totally endoscopic robotic mitral valve repair in a patient with severe mitral regurgitation. The mitral valve was repaired robotically by standard techniques, and the intraoperative transesophageal echocardiogram demonstrated no residual mitral regurgitation. However, there was unexpected hypokinesia of the posterior and lateral walls of the left ventricle, with subsequent electrocardiography showing acute ST elevations of the lateral segment. Immediate cardiac catheterization revealed occlusion of the left circumflex artery. Aspiration thrombectomy was performed and a drug-eluting stent placed to restore the contour, thus preventing potential morbidity of the patient.

Iatrogenic left circumflex artery (LCX) injury is a rare but well-documented complication of mitral surgery. Common mechanisms of this injury include entrapment with an encircling valve or annuloplasty suture, obliteration by a suture through and through the artery lumen, thrombosis due to laceration, external compression by the sutured annuloplasty ring, and vessel kinking due to annular plication.^1^^,^^2^ Early recognition and intervention are imperative to minimize adverse outcomes. Increased rates of morbidity and mortality are observed in patients needing postoperative percutaneous coronary intervention (PCI) after mitral valve repair (MVR).^3^ Moreover, postoperative PCI occurs more frequently after MVR compared with aortic valve repair, possibly because of the anatomic relationship between the LCX and mitral valve annulus, which can be variable in distance and may correlate with dominant circulation patterns.^3^^,^^4^

In this case report, we describe a patient undergoing robotic MVR who exhibited left dominant circulation with an LCX that was noted to course out of the atrioventricular groove and to hug the high wall of the left atrium. This abnormal variation predisposed him to LCX injury that was subsequently addressed by catheterization techniques. Prompt recognition of these injuries is paramount in preventing potential morbidity and mortality.

A 75-year-old man with a long-standing history of severe mitral regurgitation presented with worsening shortness of breath and dyspnea on exertion. Transthoracic echocardiography demonstrated severe mitral regurgitation with a flail posterior mitral leaflet. Preoperative cardiac catheterization revealed minimal coronary artery disease and a dominant LCX (Figure 1). The patient underwent a totally endoscopic robotic MVR. Intraoperative findings confirmed significant P2 prolapse. There was no excessive leaflet tissue, and the disease process was consistent with fibroelastic deficiency. The left atrial appendage was sutured closed in a 2-layer extended fashion (Video). We reduced the atrial size by plicating the atrial vestibule between the posterior aspect of the mitral annulus and the inferior pulmonary vein.^5^ Ruptured chordae were débrided, and the flail segment was resupported with 2 pairs of artificial chordae. In addition, a P2-P3 cleft was closed and the repair completed with a 32-mm semirigid band annuloplasty. The patient was weaned from cardiopulmonary bypass without inotropic support.

**Figure 1:**
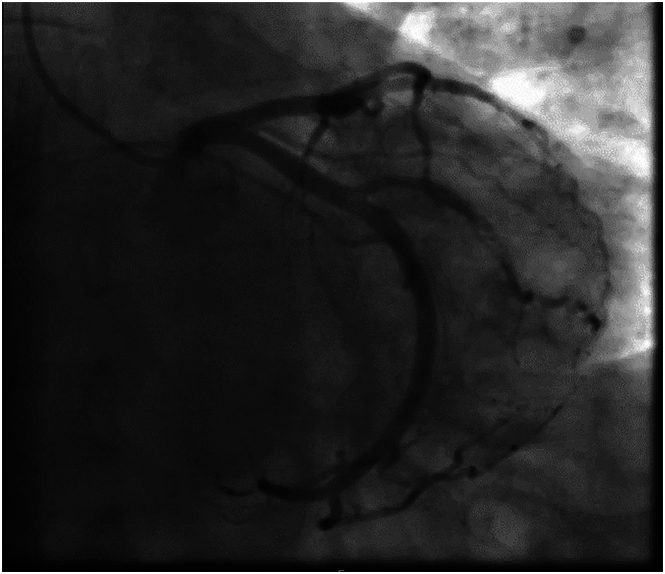
Preoperative cardiac catheterization showing patent left circumflex coronary artery.

After bypass, intraoperative transesophageal echocardiography revealed good repair without residual mitral regurgitation; however, the posterior and lateral walls of the left ventricle were hypokinetic. Electrocardiography revealed ST elevations in the lateral segments (Figure 2). The patient was rapidly moved into a hybrid operating room where cardiac catheterization revealed occlusion of the distal LCX distant from the mitral annulus (Figure 3). The LCX was noted to have an abnormal course, traveling 2.0 cm above the annulus (toward the atrium) and coursing behind the left atrial wall, confirmed on preoperative computed tomography imaging with 3-dimensional reconstruction (Supplemental Figure). We hypothesize that the plication suture in the posterior atrial wall during vestibular reduction created a mass effect and squeezed the abnormal LCX. Aspiration thrombectomy was performed, and a drug-eluting stent restored the contour, resulting in resolution of the electrocardiographic changes. Repeated transthoracic echocardiography on postoperative day 2 showed normal left ventricular wall motion. Postoperative electrocardiography displayed sinus rhythm with left axis deviation. The patient was discharged home on postoperative day 3. At 1-year follow-up, the patient endorsed good exercise tolerance with no complaints.

**Figure 2:**
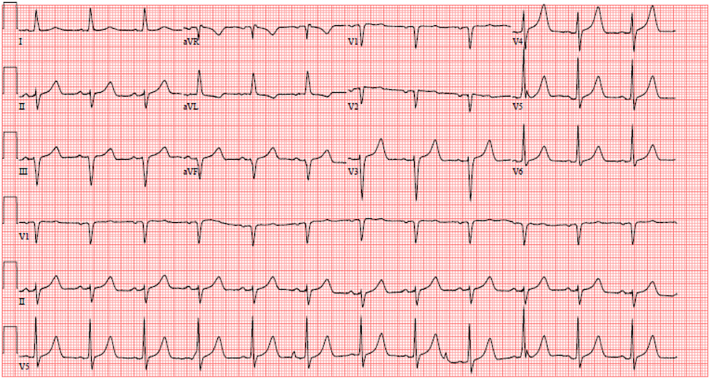
Postoperative electrocardiography demonstrating ST elevations in the lateral segments. (aVR, augmented vector right; aVL, augmented vector left; aVF, augmented vector foot.)

**Figure 3:**
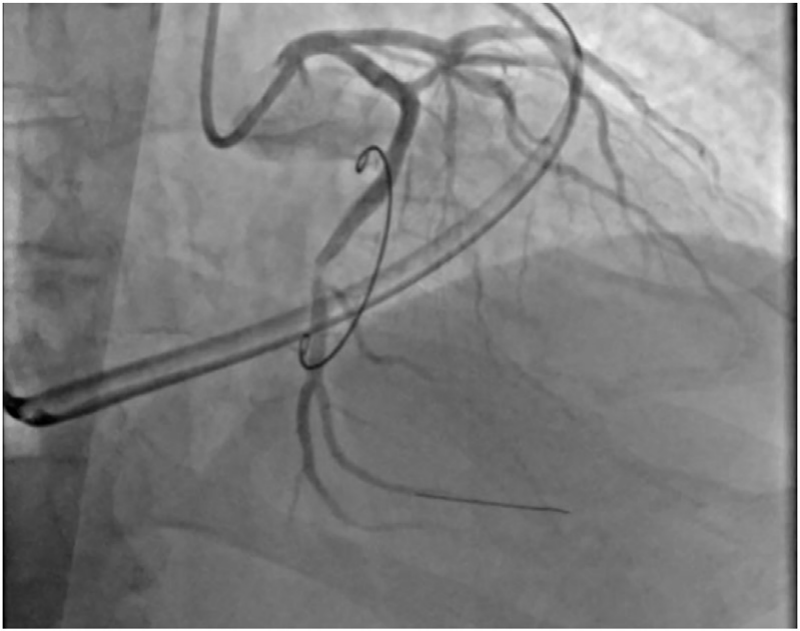
Postoperative cardiac catheterization showing the occluded left circumflex coronary artery.

## Comment

Postoperative coronary ischemia due to iatrogenic coronary injury after valve repair is a rare occurrence but has severe morbidity implications if it is not managed promptly and appropriately. In a study by Alqahtani and colleagues^3^ of >100,000 patients who underwent mitral repair or replacement, 0.8% required postoperative coronary angiography and PCI before discharge. The unadjusted in-hospital mortality rate was significantly higher in patients who had PCI compared with those who did not (22.4% vs 5.5%; *P* < .001). Moreover, the rate of postoperative PCI was higher after isolated MVR (1.15%) compared with isolated aortic valve replacement (0.51%), probably attributed to the relationship between the course of the LCX and the mitral valve annulus.

The LCX normally travels posteriorly and intimately along the mitral valve annulus, with varying distances from the atrioventricular groove.^1^ Interestingly, studies have reported differences in the distance of the LCX above the mitral annulus in patients with right dominant, codominant, or left dominant circulation patterns. In more than three-quarters of patients, the posterior descending artery originates from the right coronary artery, defined as right dominant circulation. Left dominant circulation, in which the posterior descending artery emerges from the LCX, exists in 8% of patients.^4^ Virmani and colleagues^4^ observed a closer distance between the LCX and mitral annulus in hearts with left dominant circulation. They found a distance between the LCX and mitral annulus of 4.1 mm in left dominant compared with 5.5 mm in codominant and 8.4 mm in right dominant circulation. With these findings, there may be a theoretical increased risk of LCX injury during MVR in patients with left dominant circulation.

In this case, the patient was noted to have an abnormal course of the LCX that traveled outside of the atrioventricular groove and high along the left atrium. Perhaps noncoincidentally, the patient also had left dominant circulation. It is likely that during our plication of the atrial vestibule,^5^ there was inadvertent entrapment or compression of the LCX that led to the subsequent regional ischemic changes, noted immediately on transesophageal echocardiography and electrocardiography. Whereas it could be argued that prompt sternotomy, cardiopulmonary bypass, and blind, targeted bypass grafting could have been performed, the immediate availability of a hybrid operating room and the patient’s stability allowed precise definition of the anatomic issue and immediate correction. We maintain that thorough preoperative evaluation and intraoperative care can mitigate the risk of this rare but serious complication.

## Figures and Tables

**Supplemental Figure:**